# Challenging the *status quo*: a scoping review of value-based care models in cardiology and electrophysiology

**DOI:** 10.1093/europace/euae210

**Published:** 2024-08-19

**Authors:** Lucia Osoro, Maura M Zylla, Frieder Braunschweig, Francisco Leyva, Josep Figueras, Helmut Pürerfellner, Josè Luis Merino, Ruben Casado-Arroyo, Giuseppe Boriani

**Affiliations:** Department of Cardiology, H.U.B.-Hôpital Erasme, Université Libre de Bruxelles, Rte de Lennik 808, 1070 Bruxelles, Belgium; Department of Cardiology, HCR (Heidelberg Center for Heart Rhythm Disorders), Medical University Hospital, Im Neuenheimer Feld 410, 69120 Heidelberg, Germany; mHealth and Health Economics and PROM Committee of EHRA (European Heart Rhythm Association), Rue de la Loi 34/6th Floor B, 1040 Bruxelles, Belgium; mHealth and Health Economics and PROM Committee of EHRA (European Heart Rhythm Association), Rue de la Loi 34/6th Floor B, 1040 Bruxelles, Belgium; Department of Medicine, Solna Karolinska Institutet and ME Cardiology, Karolinska University Hospital, Norrbacka S1:02, Eugeniavagen 27, Stockholm 171 77, Sweden; mHealth and Health Economics and PROM Committee of EHRA (European Heart Rhythm Association), Rue de la Loi 34/6th Floor B, 1040 Bruxelles, Belgium; Department of Cardiology, Aston Medical Research Institute, Aston Medical School, Aston University, Aston Triangle, Birmingham B4 7ET, UK; European Observatory of Health Systems and Policies, Place Victor Horta 40/30 Eurostation, 1060 Brussels, Belgium; mHealth and Health Economics and PROM Committee of EHRA (European Heart Rhythm Association), Rue de la Loi 34/6th Floor B, 1040 Bruxelles, Belgium; Ordensklinikum Linz Elisabethinen, Interne II/Kardiologie und Interne Intensivmedizin, Fadingerstraße 1, 4020 Linz, Austria; mHealth and Health Economics and PROM Committee of EHRA (European Heart Rhythm Association), Rue de la Loi 34/6th Floor B, 1040 Bruxelles, Belgium; Arrhythmia-Robotic Electrophysiology Unit, La Paz University Hospital, IdiPAZ, Universidad Autónoma, Madrid, Spain; Department of Cardiology, H.U.B.-Hôpital Erasme, Université Libre de Bruxelles, Rte de Lennik 808, 1070 Bruxelles, Belgium; mHealth and Health Economics and PROM Committee of EHRA (European Heart Rhythm Association), Rue de la Loi 34/6th Floor B, 1040 Bruxelles, Belgium; mHealth and Health Economics and PROM Committee of EHRA (European Heart Rhythm Association), Rue de la Loi 34/6th Floor B, 1040 Bruxelles, Belgium; Cardiology Division, Department of Biomedical, Metabolic and Neural Sciences, University of Modena and Reggio Emilia, Policlinico di Modena, Via del Pozzo, 71, Modena 41124, Italy

**Keywords:** Value-based healthcare, Value-based care, Electrophysiology, Cardiology

## Abstract

**Aims:**

The accomplishment of value-based healthcare (VBHC) models could save up to $1 trillion per year for healthcare systems worldwide while improving patients’ wellbeing and experience. Nevertheless, its adoption and development are challenging. This review aims to provide an overview of current literature pertaining to the implementation of VBHC models used in cardiology, with a focus on cardiac electrophysiology.

**Methods and results:**

This scoping review was conducted according to the Preferred Reporting Items for Systematic Reviews and Meta-analysis for Scoping Reviews. The records included in this publication were relevant documents published in *PubMed*, *Mendeley*, and *ScienceDirect*. The search criteria were publications about VBHC in the field of cardiology and electrophysiology published between 2006 and 2023. The implementation of VBHC models in cardiology and electrophysiology is still in its infant stages. There is a clear need to modify the current organizational structure in order to establish cross-functional teams with the patient at the centre of care. The adoption of new reimbursement schemes is crucial to moving this process forward. The implementation of technologies for data analysis and patient management, among others, poses challenges to the change process.

**Conclusion:**

New VBHC models have the potential to improve the care process and patient experience while optimizing the costs. The implementation of this model has been insufficient mainly because it requires substantial changes in the existing infrastructures and local organization, the need to track adherence to guidelines, and the evaluation of the quality of life improvement and patient satisfaction, among others.

## Graphical Abstract

**Graphical abstract:**
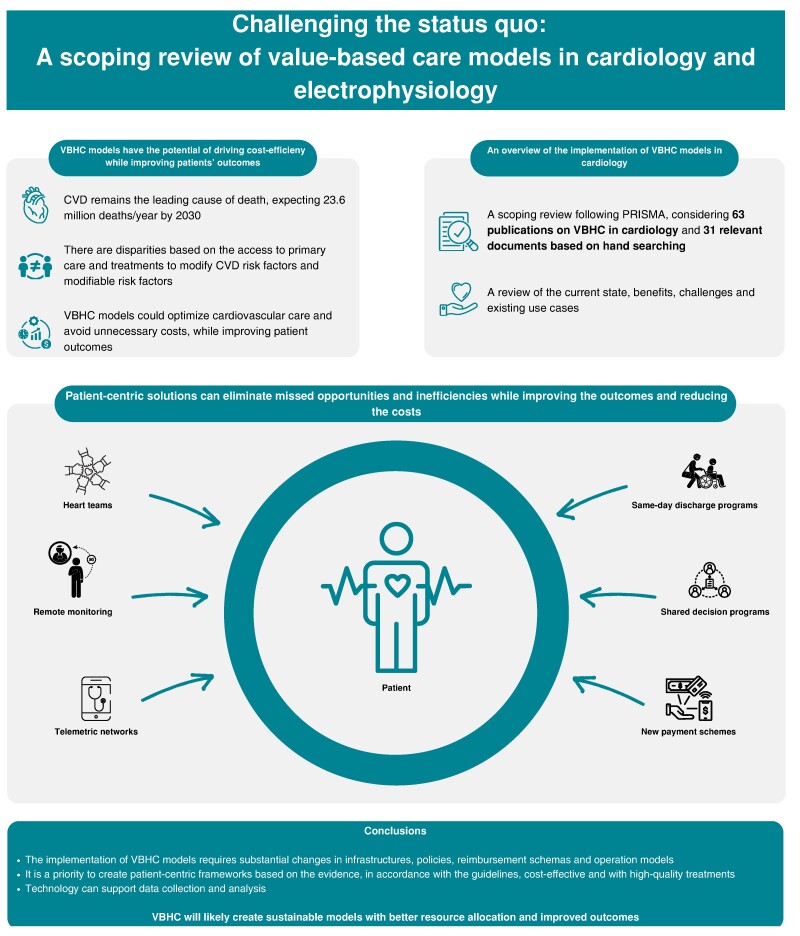

## Table of contents

1. Introduction
2. Methods
3. Results
  3.1. VBHC: new models for value maximization
  3.2. Existing solutions to be implemented in cardiology
  3.3. New cross-functional teams with the patient at the centre
  3.4. Policy levers to support VBHC models
  3.5. Payment models that incentivize the health outcomes and quality of care
  3.6. The pioneers of VBHC in Europe
  3.7. Challenges to adopt VBHC models in cardiology
4. Discussion
  4.1. Limitations
5. Conclusion
Funding
Data availability

**What’s new ?**

- VBHC models at macro level have been widely discussed, but its implementation in cardiology is in its infant stages.
- The scoping review outlines the current status of VBHC models in cardiology.
- During the review, the authors have identified some projects that create patient-centric cardiology clinics and boost the implementation of VBHC model.

## 1. Introduction

Cardiovascular diseases (CVDs) remain the leading cause of death, accounting for 17.3 million deaths globally, and it is expected to account for more than 23.6 million deaths per year by 2030.^1^ According to the American Heart Association, the decline in the mortality trends seems to be dissipating, and while CVD mortality has remained stable (0.5% per year), other diseases such as cancer have continued to decrease. Chronic conditions like heart failure have reached a plateau phase in terms of mortality decrease, reflecting no change since 2015. This plateau phase is also affecting stroke mortality with few changes since 2017. Most importantly, there are disparities based on the access to primary care and treatments to modify cardiovascular risk factors and modifiable risk factors.^2^

The existing care pathways and schemes present multiple missed opportunities that could improve cardiovascular care and avoid unnecessary costs. Solutions related to risk factor modifications, patient engagement and involvement, accurate diagnosis, adherence to treatments, adequate use of advanced treatments, and efficient use of support services would contribute to cost reduction and optimized care delivery.^2,3^

Additionally, the traditional payment for health services in many countries is based on fee-for-service models. These fee-for-service models compensate healthcare providers based on the volume of services they deliver, such as the number of procedures, tests, or in-office visits, rather than the outcomes or the value delivered to the patients. This payment scheme can sometimes lead to overutilization of healthcare services and may not always be aligned with the best interests of patients in terms of quality and cost-efficiency.^4^

There is a general consensus that current payment models are not sufficient for addressing global challenges in health care. These payment models might not adequately deal with ageing populations and rising levels of multimorbidity that increase the demand for care services. This situation reflects the traditional healthcare models, which incentivize the volume of patients and procedures, over the outcomes for the patients.^3–7^

**Figure 1:**
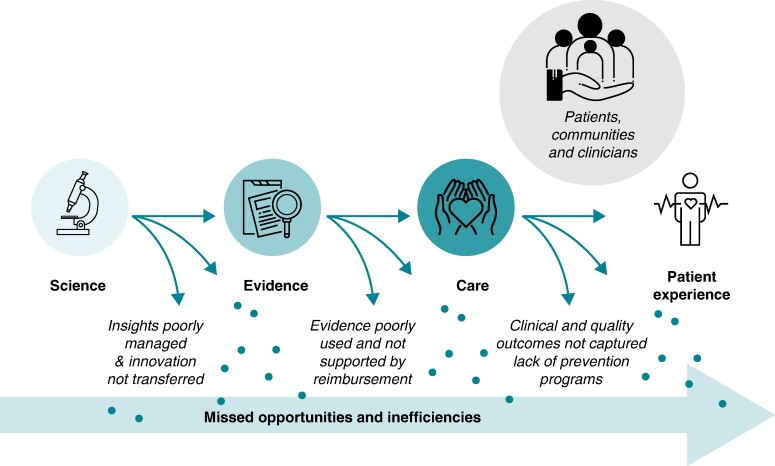
Disconnected healthcare system with missed opportunities and inefficiencies (modified from ‘Introduction to high-value care, EIT Health’^14^).

In 2006, Michael Porter and Elizabeth Teisberg redefined the concept of value in healthcare, in their seminal book *Redefining Health Care*, as the outcomes that matter to patients divided by the cost to achieve these outcomes.^8^ Besides considering value-based care as a mathematical formula that could help quantify the impact in a clinical unit setup, the whole concept of value-based healthcare (VBHC) or value-based care seeks the improvement of societal wellbeing with the patient at the centre of care. The use of non-patient-centric healthcare models that do not seek the best outcomes with optimized costs has an impact of $1 trillion globally per year, on the basis of the inherent inefficiencies of healthcare systems (*Figure 1*).^5,9–13^

Over time, new challenges have been raised for healthcare leaders. Some examples are the need to connect quality of life and quality of care services, the development of new ways of working while renovating and improving the existing solutions, the prioritization of the vulnerable members of society, the demand to redefine the concept of solidarity, and the requirement to include healthcare knowledge in the policy system. This new landscape, which political and healthcare leaders are facing, is renewing the emphasis on improving patient outcomes, with an increased concern about practice variations in the standards of care delivery and growing awareness of gaps in safety, efficiency, and person-centredness.^11,13,15^

In the past decades, clinical cardiology has gradually evolved into a highly specialized discipline, notably exemplified by modern electrophysiology—a branch utilizing advanced medical technology with a focus on effective invasive procedures.

Although the progressive subspecialization in cardiology has led to remarkable advancements in patient survival and wellbeing, it has also been linked to adverse effects such as a fragmentation of care with ‘siloed’ specialized units, a lack of multidisciplinary integration, the loss of a holistic care approach, and escalating costs.^16^ VBHC has been suggested to overcome these drawbacks. Therefore, our review aims to better understand the concept of VBHC, its potential strengths, and the challenges of applying this model in cardiology and electrophysiology. Moreover, this review, driven by the Committee on Health Economics of the European Heart Rhythm Association (EHRA), aims to provide an overview of the different projects of VBHC adoption in the context of cardiology and more specifically in electrophysiology, where a holistic approach could potentially address challenges such as patient engagement and involvement, improvements in diagnosis, adherence to treatments, or efficient support of services that could avoid unnecessary costs and contribute to the modification and reduction in risks factors.^17^

## 2. Methods

**Figure 2:**
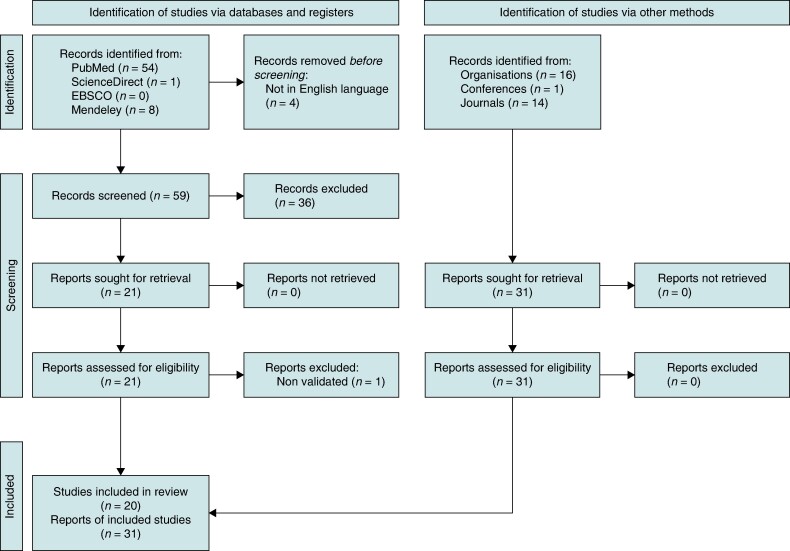
PRISMA 2020 flow diagram for new systematic reviews which included searches of databases, registers, and other sources.

For the purpose of this scoping review, we considered 63 main relevant publications on the subject of VBHC. We followed the PRISMA research flow (*Figure 2*) with the intention of studying the implementation of this model in the field of cardiology and electrophysiology. Four databases were used: *PubMed* (*n* = 54), *ScienceDirect* (*n* = 1), *EBSCO* (*n* = 0), and *Mendeley* (*n* = 8). The terms used for the search were: ‘value-based healthcare’, ‘VBHC’, ‘cardiology’, and ‘electrophysiology’. We considered publications between 2006 and 2023 in English, French, Italian, and Spanish. With these considerations, 59 publications out of the 63 were considered for the initial screening (*Table 1*).

**Table 1:** Identification, screening, and inclusion table

	Identification	Screening			Inclusion
	Paper (syst. review) (name, year)	VBHC (Y/N)	Patient-centric solution (Y/N)	Validated Study (Y/N)	To be included (Y/N)
1	J.C. Ray et al, 2016—The transition to value-based care	Y	Y	Y	Y
2	D.S. Chew et al, 2022—Cost-effectiveness of catheter ablation versus antiarrhythmic drug therapy in atrial fibrillation: the CABANA randomized clinical trial	N	N	Y	N
3	H.Yogasundaram et al, 2021—Cardiomyopathies and genetic testing in heart failure: role in defining phenotype-targeted approaches and management	N	N	Y	N
4	K.T.Nguyen et al, 2017—Smartphone-based geofencing to ascertain hospitalizations	N	Y	Y	N
5	S. M. Kim et al, 2019—Public reporting on cardiac electrophysiology procedures and outcomes: where are we now and where are we headed?	Y	Y	Y	Y
6	A.N.L. Hermans et al, 2020—On-demand mobile health infrastructures to allow comprehensive remote atrial fibrillation and risk factor management through teleconsultation	N	Y	Y	Y
7	D. Slotwiner, 2016—Electronic health records and cardiac implantable electronic devices: new paradigms and efficiencies	N	Y	Y	Y
8	W. H. Seligman et al, 2020—Development of an international standard set of outcome measures for patients with atrial fibrillation: a report of the International Consortium for Health Outcomes Measurement (ICHOM) atrial fibrillation working group	Y	N	Y	Y
9	J. Schweber et al, 2022—Implementation and early experience of a pediatric electrophysiology telehealth program	N	N	Y	N
10	D. Slotwiner et al, 2013—Cost efficiency and reimbursement of remote monitoring: a US perspective	N	Y	Y	Y
11	G. Boriani et al, 2018—Battery longevity of implantable cardioverter-defibrillators and cardiac resynchronization therapy defibrillators: technical, clinical and economic aspects. An expert review paper from EHRA	N	Y	Y	Y
12	S.Pecha et al, 2013—Concomitant surgical atrial fibrillation ablation and event recorder implantation: better monitoring, better outcome?	N	Y	Y	Y
13	H-Heger et al, 2022—Phrenic nerve injury during cryoballoon-based pulmonary vein isolation: results of the worldwide YETI registry	N	N	Y	N
14	S.Schnaubelt et al, 2023—Arterial stiffness in acute coronary syndrome as a potential triage tool: a prospective observational study	N	N	Y	N
15	Q.Cheng et al, 2020—Inhibitory effects of cyclopiazonic acid on the pacemaker current in sinoatrial nodal cells	N	N	Y	N
16	S. Ceresnak et al. 2016—Pediatric cardiology boot camp: description and evaluation of a novel intensive training program for pediatric cardiology trainees	N	N	Y	N
17	S. Störk et al, 2022—Pulmonary artery sensor system pressure monitoring to improve heart failure outcomes (PASSPORT-HF): rationale and design of the PASSPORT-HF multicenter randomized clinical trial	Y	Y	Y	Y
18	C. Tripp et al, 2020—Physical activity in adults with wearable cardioverter defibrillators in the post-myocardial infarction period	N	N	N	N
19	A. Gruber et al, 2022—Optogenetic control of human induced pluripotent stem cell-derived cardiac tissue models	N	N	Y	N
20	A. Metzner et al, 2022—What we have learned: is pulmonary vein isolation still the cornerstone of atrial fibrillation ablation?	N	N	Y	N
21	B. Wallace et al, 2021—Development and piloting of four decision aids for implantable cardioverter-defibrillators in different media formats	Y	Y	N	Y
22	I. García-Bolao et al, 2022— Local impedance drop predicts durable conduction block in patients with paroxysmal atrial fibrillation	N	N	Y	N
23	E. Baldi et al, 2020—An Utstein-based model score to predict survival to hospital admission: the UB-ROSC score	N	N	Y	N
24	G.L. Botto et al, 2022—The value of wearable cardioverter defibrillator in adult patients with recent myocardial infarction: economic and clinical implications from a health technology assessment perspective	Y	Y	Y	Y
25	S. M. Werhahn et al, 2022—NT-proBNP as a marker for atrial fibrillation and heart failure in four observational outpatient trials	N	N	Y	N
26	R. Godin et al, 2019—Screening for atrial fibrillation using a mobile, single-lead electrocardiogram in Canadian primary care clinics	Y	Y	N	N
27	A. Asundi et al, 2020—Development and validation of a semi-automated surveillance algorithm for cardiac device infections: insights from the VA CART program	N	N	Y	N
28	Z. H. Tseng et al, 2018—Prospective countywide surveillance and autopsy characterization of sudden cardiac death: POST SCD study	N	N	Y	N
29	F. Giannetti et al, 2023—Gene- and variant-specific efficacy of serum/glucocorticoid-regulated kinase 1 inhibition in long QT syndrome types 1 and 2	N	N	N	N
30	C. Schmidt et al, 2015—Upregulation of K(2P)3.1 K + current causes action potential shortening in patients with chronic atrial fibrillation	N	N	Y	N
31	B.J. Selim et al, 2016—The association between nocturnal cardiac arrhythmias and sleep-disordered breathing: the DREAM study	N	N	N	N
32	A. Krishnaswamy et al, 2020—The utility of rapid atrial pacing immediately post-TAVR to predict the need for pacemaker implantation	N	N	Y	N
33	A. Piro et al, 2020—Management of cardiac implantable electronic device follow-up in COVID-19 pandemic: lessons learned during Italian lockdown	Y	Y	Y	Y
34	B. Müller-Edenborn et al, 2022—Determinants of fibrotic atrial cardiomyopathy in atrial fibrillation. A multicenter observational study of the RETAC (reseau européen de traîtement d'arrhythmies cardiaques)-group	Y	N	Y	Y
35	P. Münkler et al, 2018—Ablation index for catheter ablation of atrial fibrillation—clinical applicability and comparison with force-time integral	N	N	Y	N
36	O. Klein-Wiele et al, 2016—A novel cross-sector telemedical approach to detect arrhythmia in primary care patients with palpitations using a patient-activated event recorder	N	Y	Y	Y
37	A. Asundi et al, 2019—Real-world effectiveness of infection prevention interventions for reducing procedure-related cardiac device infections: Insights from the Veterans Affairs clinical assessment reporting and tracking program	N	N	Y	N
38	K. Deutsch et al, 2015—Validation of standard and new criteria for the differential diagnosis of narrow QRS tachycardia in children and adolescents	N	N	Y	N
39	B. G. Aktaa et al, 2023—Data standards for atrial fibrillation/flutter and catheter ablation: the European unified registries for heart care evaluation and randomized trials (EuroHeart)	Y	N	Y	Y
40	L. Gao et al, 2020—Can combined screening of ultrasound and elastography improve breast cancer identification compared with MRI in women with dense breasts-a multicenter prospective study	N	N	Y	N
41	C. Eickholt et al, 2018—Sympathetic and parasympathetic coactivation induces perturbed heart rate dynamics in patients with paroxysmal atrial fibrillation	N	N	Y	N
42	A. Kurek et al, 2017—Impact of remote monitoring on long-term prognosis in heart failure patients in a real-world cohort: results from all-comers COMMIT-HF trial	Y	Y	Y	Y
43	L. Marzec et al, 2018—Device-measured physical activity data for classification of patients with ventricular arrhythmia events: A pilot investigation	N	N	N	N
44	L. Frigerio et al, 2021—End-tidal carbon dioxide (ETCO2) and ventricular fibrillation amplitude spectral area (AMSA) for shock outcome prediction in out-of-hospital cardiac arrest. Are they two sides of the same coin?	N	N	Y	N
45	P. Amedro et al, 2023—Use of new paediatric VO_2max_ reference equations to evaluate aerobic fitness in overweight or obese children with congenital heart disease	N	N	Y	N
46	L. Jian et al, 2023—Association between albumin corrected anion gap and 30-day all-cause mortality of critically ill patients with acute myocardial infarction: a retrospective analysis based on the MIMIC-IV database	N	N	Y	N
47	G. Boriani et al, 2021—Cost-minimization analysis of a wearable cardioverter defibrillator in adult patients undergoing ICD explant procedures: Clinical and economic implications	Y	Y	Y	Y
48	F. Ahmed et al, 2021—Use of healthcare claims to validate the prevention of arrhythmia device infection trial cardiac implantable electronic device infection risk score	N	N	Y	N
49	B.Wallace et al, 2020—A multicenter trial of a shared decision support intervention for patients offered implantable cardioverter-defibrillators: DECIDE-ICD rationale, design, Medicare changes, and pilot data	Y	Y	Y	Y
50	C. Godino et al, 2020—Inappropriate dose of nonvitamin-K antagonist oral anticoagulants: prevalence and impact on clinical outcome in patients with nonvalvular atrial fibrillation	N	N	Y	N
51	G. Vetta et al, 2023—The r'-Wave Algorithm: a new diagnostic tool to predict the diagnosis of Brugada syndrome after a sodium channel blocker provocation test	N	N	Y	N
52	D. Mehta et al, 2008—Cardiac involvement in patients with sarcoidosis: diagnostic and prognostic value of outpatient testing	N	N	Y	N
53	M.J.P. Raatikainen et al, 2015—Current trends in the use of cardiac implantable electronic devices and interventional electrophysiological procedures in the European Society of Cardiology member countries: 2015 report from the European Heart Rhythm Association	Y	N	Y	Y
54	N. Lowres et al, 2019—Estimated stroke risk, yield, and number needed to screen for atrial fibrillation detected through single time screening: a multicountry patient-level meta-analysis of 141 220 screened individuals	N	N	Y	N
55	E. Wyffels et al, 2023—Same day discharge strategy by default in a tertiary catheterization laboratory in Belgium: value based healthcare-change in practice	Y	Y	Y	Y
56	S. Adzic et al, 2009—The public healthcare system in the transition countries the case study of Serbia	N	N	Y	N
57	M. Ghani et al, 2014—Enhancing service delivery in FM: case study of a Malaysian healthcare facilities directorate	N	N	Y	N
58	A. Behrend et al, 2015—Mastering situation awareness in healthcare database systems	N	N	Y	N
59	E. Ziemba et al, 2011—Importance and impact of ERP systems on industry and organization	N	N	N	N

The use of the terms ‘VBHC’ or ‘value-based healthcare’ excluded some publications focused on effectiveness or quality that could have been relevant to the research. Nevertheless, none of them was specific to electrophysiology or cardiology, as those terms were also used during the research. Therefore, its inclusion would arguably add value to the focus of our research and would complicate the revision of the literature.

The inclusion criteria used during the initial screening considered papers that address and support the results stated in their objectives and met at least one out of the following criteria: explicit application of the VBHC model in the publication or description and/or inclusion of any patient-centric solution. Following this criteria, 20 publications were considered after the screening phase.

To further support our research, we also considered 31 relevant documents in the field of VBHC and cardiology based on hand searching. These publications were key documents by the World Health Organization, World Economic Forum, European Society of Cardiology (ESC), American Heart Association, EIT Health, American Medical Association, Harvard Business School, and European Observatory of Health Systems and Policies, providing guidelines for healthcare and VBHC adoption and relevant publications on the field of cardiology and electrophysiology (*Table 2*). Thus, our search resulted in 51 documents considered for this scoping review.

**Table 2:** Reference publications to further support our research with a focus on VBHC, cardiology, and electrophysiology

Year	Authors	Study name
2015	IEEE Conference	Mastering situation awareness in healthcare database systems
2020	European Health Management Association	EHMA 2020: Conference Report
2019	Busse, Reinhard, Dimitra Panteli, and Wilm Quentin	*Improving healthcare quality in Europe characteristics, effectiveness and implementation of different strategies: characteristics, effectiveness and implementation of different strategies, chapter 1*
2016	The Economist Intelligence Unit	Value-based healthcare: a global assessment
2022	EIT Health	Introduction to high value care
2021	EIT Health	Implementing value-based health care in Europe: Handbook for pioneers
2013	European Society of Cardiology	The heart team to assess risk in coronary artery disease an article from the e-Journal of the ESC Council for Cardiology Practice
2018	European Society of Cardiology	2018 ESC/EACTS guidelines on myocardial revascularization
2016	European Society of Cardiology	Das herzteam bei der planung und durchführung von revaskularisationen: ESC-leitlinien versus klinischer alltag
2010	European Society of Cardiology	Task force on myocardial revascularization of the European Society of Cardiology (ESC) and the European Association for Cardio-Thoracic Surgery (EACTS)
2022	European Society of Cardiology	2021 ESC/EACTS guidelines for the management of valvular heart disease
2021	Krohwinkel, Anna, Unni Mannerheim, Jon Rognes, and Hans Winberg	Value-based healthcare in theory and practice. What have we learned? Lessons from the Swedish experience
2006	Porter, Michael, and Elizabeth Teisberg	Redefining health care: creating value-based competition on results
2023	World Health Organization	Building on value-based health care: towards a health system perspective
2020	World Health Organization	Building on value-based healthcare. towards a health system perspective
2012	Institute for Healthcare Improvement	A guide to measuring the triple aim: population health, experience of care, and per capita cost
2018	Stokes, Jonathan, Verena Struckmann, Søren Rud Kristensen, Sabine Fuchs, Ewout Van Ginneken, Apostolos Tsiachristas, Maureen Rutten Van Mölken, and Matt Sutton	Towards incentivising integration: a typology of payments for integrated care
2017	World Economic Forum	Laying the foundation for health system transformation
2021	World Health Organization	Fact sheet: cardiovascular diseases (CVDs)
2021	World Health Organization	From value for money to value-based health services: a twenty-first century
2023	World Health Organization	Universal health coverage
2019	American Heart Association	Call to action: urgent challenges in cardiovascular disease: a presidential advisory from the American Heart Association
2023	Stevenson, Lynne Walter	Remote monitoring for heart failure management at home
2022	Boriani G, Svennberg E, Guerra F, Linz D, Casado-Arroyo R, Malaczynka- Rajpold K, Duncker D, Boveda S, Merino JL, Leclercq C.	Reimbursement practices for use of digital devices in atrial fibrillation and other arrhythmias: a European Heart Rhythm Association survey
2022	Boriani G, Vitolo M, Svennberg E, Casado-Arroyo R, Merino JL, Leclercq C.	Performance-based risk-sharing arrangements for devices and procedures in cardiac electrophysiology: an innovative perspective
2022	Boriani G,Burr H, Svennberg E, Imberti JF, Merino JL, Leclercq C	Current status of reimbursement practices for remote monitoring of cardiac implantable electrical devices across Europe
2023	Varma N, Braunschweig F, Burri H, Hindricks G, Linz D, Michowitz Y, Ricci RP, Nielsen JC	Remote monitoring of cardiac implantable electronic devices and disease management
2023	Ferrick AM *et al.*	2023 HRS/EHRA/APHRS/LAHRS expert consensus statement on practical management of the remote device clinic
2023	Boriani G, Imberti J, Leyva F, Casado-Arroyo R, Chun J, Braunschweig, F, Zylla M, Duncker D, Farkowski M, Pürerfellner H, Merino JL	Length of hospital stay for elective electrophysiological procedures: a survey from the European Heart Rhythm Association
2023	Januszkiewicz L, Barra S, Marijon E, Providencia R, De Asmundis C, Chun J, Farkowski M, Conte G, Boveda S.	Major gaps in the information provided to patients before implantation of cardioverter defibrillators: a prospective patient European evaluation
2023	Gabriels J, Schaller R, Koss E, Rutkin B, Carrillo R, Epstein L	Lead management in patients undergoing percutaneous tricuspid valve replacement or repair: a ‘heart team’ approach

## 3. Results

Of the included papers, 90% were published within the past 10 years. Thirty-five per cent (7 out of 20) were written by US authors, based on the affiliation of the first author and 20% (4 out of 20) by German researchers. During the review, the USA has been identified as the first country to adopt and implement VBHC models across its healthcare system. Out of the 20 publications, 20% (4 out of 20) were randomized trials, and 10% (2 out of 20) were a systematic review of the literature. Five per cent (1 out the 20) was a single-centre study including over 10 000 patients.

Our review comprehends information on the relevance of VBHC models and their potential, those existing solutions that have been adopted in cardiology according to VBHC models, the creation of cross-functional teams with the patient at the centre of care, new policy levers that support VBHC implementation, the payment models and schemes that would incentivize the value of care rather than the traditional fee-per-service models, the experience of the early adopters of these models in Europe, and the challenges that VBHC implementation would present in the context of cardiology and electrophysiology.

### 3.1. VBHC: new models for value maximization

**Figure 3:**
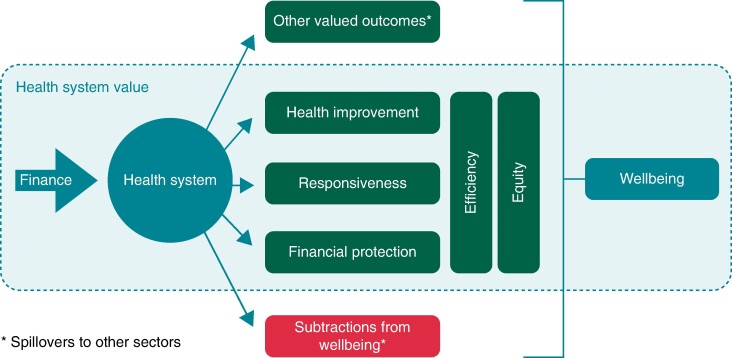
Value from a health system perspective. Source: Smith PC, Sagan A, Siciliani L, et al. Building on value-based health care: Towards a health system perspective. Copenhagen (Denmark): WHO Regional Office for Europe on behalf of the European Observatory on Health Systems and Policies (Policy Brief, No. 37.)

A recent revision of the traditional VBHC model, developed by Porter and Teisberg in 2006, has led to a new definition of value for the healthcare system. The expectation from a VBHC model is to convert the funds allocated for care into the improvement of societal wellbeing (*Figure 3*).^9^

These VBHC models have the potential of maximizing societal wellbeing, while eliminating inefficiencies within the overall healthcare expenditure estimated from 20% to 40%.^18^ The focus and ultimate goal of its implementation is to create value to the healthcare system.^9^

According to the latest objectives developed by the World Health Organization, the central priorities of any healthcare system should be health improvement, responsiveness, financial protection, efficiency, and equity, and these goals should reflect the health system's concept of value. Concepts such as efficiency and equity are to be included as transversal objectives of this model. In the concept of efficiency, the authors focus on the delivery of care with minimal resources, avoiding unnecessary treatments and consultations, and equity relates to the distribution of care services across the entire population.^9^

### 3.2. Existing solutions to be implemented in cardiology

There are very diverse patient-centred solutions that have been shown to improve the outcomes for arrhythmia patients and their quality of life. These solutions are initiatives that have been individually deployed as efforts embedded in the need to create value within the context of the traditional healthcare systems and contribute to addressing some challenges in the context of the arrhythmia unit with the spirit of improving the outcomes and lowering the overall costs. Nevertheless, all these individual initiatives are rarely part of a coordinated VBHC strategy.^17,19^

**Figure 4:**
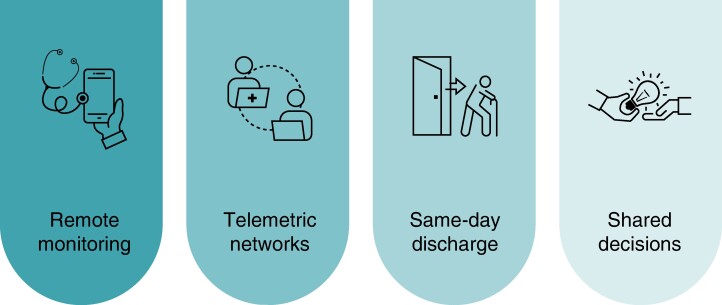
Programs to support VBHC implementation at the cardiology department.

During the revision of literature, we have identified four solutions specifically designed to implement VBHC schemas in the context of cardiovascular and integrate most of the key action points mentioned as part of the results of our findings (*Figure 4*).^20–27^

The creation of *remote monitoring* clinics allows healthcare providers to continuously monitor patients with different conditions like heart failure or arrhythmias by means of non-invasive telemonitoring or implantable electronic devices like pacemakers, ICDs, or loop recorders. The continuous surveillance of patients enables the early detection of arrhythmias, device issues, and other critical events and facilitates timely interventions for improved patient outcomes. At the same time, patient management using remote monitoring avoids unnecessary in-person visits and reduces the length of hospital stays, which contributes to cost savings.^23–25^ Remote home monitoring empowers the patients by giving them access to the measured data or enabling them to perform regular measurements themselves, offering a valuable tool to enhance person-centred care.^21,26–34^

The establishment of cross-functional *telemetric networks*, integrating primary care specialists and cardiologists, and using patient-activated event recorders to monitor patients with rhythm disorders and high risk of having a stroke has proven to improve the quality of life of these patients. Thanks to these new programs, direct oral anticoagulants were prescribed at earlier stages in cases of *de novo* AF, instead of vitamin K antagonists, with the agreement of GPs and cardiologists; cardioversion was applied in persistent AF patients; and ablation was performed in highly symptomatic patients or CIED implantation, in concordance with the guidelines.^20,22,35,36^

In the context of ablation and other invasive cardiovascular procedures, *same-day discharge programs* (SDD) in specific conditions and clinical situations have demonstrated cost savings vs. standard practice. This approach offers the convenience and comfort of returning home the same day for the patients and suggests that these programs maintain or even enhance the quality of care while optimizing healthcare resources.^31^ However, a marked variability exists with regard to the duration of hospital stay for electrophysiological procedures.^19,37,38^

*Shared decision programs* are designed to involve patients in the decision-making process and ensure they are well-informed about their medical conditions and treatment options, empowering them to make decisions aligned with their values, promoting a patient-centred approach by actively involving them and, with this active engagement, improving their satisfaction with their care, leading to higher levels of satisfaction.^28–30^

### 3.3. New cross-functional teams with the patient at the centre

The implementation of the VBHC model could be taken as a gradual process, where all the stakeholders need to be involved. It is key that every member of the organization embraces the change. As a starting point, the literature suggests that a focus on areas, where the impact could make a difference, is the best approach for the change to begin.^17,39^

The change requires new dimensions of value to be taken into consideration, and these new dimensions of value could be ultimately translated into benefits and costs while eliminating the inefficiencies in the healthcare expenditure.^17,18,40^

The value in the healthcare system is highly dependent on the different players and actors whose actions would secure the different aspects of value. The identified actors are the national policymakers, purchasers, provider organizations, practitioners, citizens, and patients (*Table 3*). The entire healthcare system would need to recognize these actors and their contributions as they impact the overall value created. The perspectives of these parties should be acknowledged in the healthcare system: the *national policymakers*, who formulate and propagate the information about the healthcare system; the *purchasers*, who plan and purchase services, acting on behalf of the national healthcare systems or hospital groups, and are in charge of ensuring a maximum value for the care services; the *provider organizations*, who are responsible for the benefits and health generated, focus on the sustainability of the financial health of the system regardless of its size, and deliver high-quality and responsive services while keeping the costs to a minimum; the *practitioners*, who contribute to the functioning of the healthcare system, both individuals and those who are part of the system, who contribute to the improvement of health for their service users; the *citizens* of the country, who contribute to the finance of the health system through tax payments or insurance premiums and who should be included in any discussion on the value of the health system; the *patients*, who are the citizens who fall ill and whose feedback on the care provided and satisfaction is key to improve the healthcare system and ensure the care provided is tailored and patient-centric.

**Table 3:** Summary of actors in the healthcare system

Actors	Value Delivered	Responsibility
1. National policymakers	Ensure all elements of the health system contribute to the concept of value defined	Formulation of a concept of value for the health system Transmission of value to all the actors Ensure the value is maximized
2. Purchasers	Health improvement, service responsiveness, and aspects of equity and efficiency	Plan and purchase services for a defined population taking into account national mandates, service and budget constraints, and legitimate variations in local health needs and contextual factors
3. Provider Organizations	Delivering high-quality and responsive services that generate health and non-health benefits, while keeping costs to a minimum	Financial sustainability, regardless of the size of the system
4. Practitioners	Improve the health of the users	Financial sustainability, regardless of the size of the system
5. Citizens	Improve the health of the users	Deliver health services while adhering to best practice guidelines and reduction of unwarranted variation
6. Patients	Improvement of the health system, contributing with their behaviour to minimize the impact of illness and maximize the benefit of treatment	Participate in discussions to ensure that the care provided is tailored to satisfy their health needs and is patient-centred

Part of the implementation of these models relies on the ability of the actors to leverage actions that would lead to an improvement in the value of healthcare, such as working across sectors for health, working on prevention programs, strengthening the primary care, improving the communication flow across specialties and primary health care, involving patients in our care, incorporating the use of new digital health tools, or setting a health benefits package, among others.^9,17,18^

The rising concept of a ‘heart team’, where a team of experts supports the patient along the whole cycle of care, has been actively promoted by the ESC^41–45^ and the American College of Cardiology (ACC).^2,46^ It supports the objectives of VBHC models. These teams contribute to the establishment of integrated, multidisciplinary care structures around patients with specific medical conditions.^2,47^ The model emphasizes the collective expertise of professionals from different medical disciplines who work together to assess, plan, and deliver comprehensive care for cardiovascular patients and, may be particularly relevant, in cases where multiple treatment modalities or interventions should be considered. 30 An example of a ‘heart team’ is exemplified in the ESC Guidelines for the Management of Valvular Heart Disease. The recommended mode of intervention in patients with aortic stenosis who are not suitable for surgical aortic valve replacement (SAVR) might be TAVI, but it should be assessed by the heart team.^45^

### 3.4. Policy levers to support VBHC models

Some examples of policy levers have been identified and discussed in the literature. In our analysis, we have identified the health in all policies (HiAP), the new fiscal and regulatory measures for health promotion and disease prevention, establishment of a universal healthcare coverage, health benefits package, strategic purchasing approach, integrated care services, evidence-based care practices, or involving the patients in their own care (*Table 4*). Efforts such as HiAP, where health considerations are to be included in the policies of other sectors, raise health awareness, advise on effective and suitable interventions, and propose collaborations: new fiscal and regulatory measures for health promotion and disease prevention, looking into mitigating those factors that can lead to a range of health problems; the establishment of a universal healthcare coverage, which seeks to finance some health services through pre-payment with risk pooling, avoiding financial barriers to the users; the health benefits package, to maximize the overall health gain, explicating the services to which citizens are entitled from publicly funded health insurances; strategic purchasing, where cost-effective services would improve the efficiency of the healthcare system; integrated care services, which would coordinate the collaboration across disciplines and orchestrate a tailored care experience for each patient; evidence-based care practices, adopting the latest scientific evidence and complying with the latest clinical guidelines; or the involvement of the patients in their own care, providing information and self-care services so patients can take an active role in the management of their health.

**Table 4:** Policy levers to promote new concepts of value. Modified from Building on value-based healthcare: towards a health system perspective, 2023^48^

Policy	Key actors	Contribution to value
Working across sectors for health: health in all policies	Ministries of health and of other sectors	Inter-sectoral co-benefits
Fiscal and regulatory measures for health promotion and disease prevention	Ministries of health and ministries of finance	Health, equity, and efficiency
Funding health care for universal access	National policymakers	Access to health services, financial protection
Setting a health benefits package	National policymakers, purchasers	Promotion of equity and efficiency
Strategic purchasing for health gain	Purchasers	Improving efficiency and quality
Integrated people-centred health services	Provider organizations, practitioners	Responsiveness, coordinated care
Evidence-based care	National policymakers, purchasers, provider organizations	Reduction in unwarranted variation, equity, quality, health
Involving patients in their own care	National policymakers, practitioners	Responsiveness, efficiency

### 3.5. Payment models that incentivize the health outcomes and quality of care

The new payment models will need to evaluate the costs of the entire care pathway and the outcomes after the entire treatment. For these results to be measured, all the partial contributions of the different actors need to be aligned. Part of this transformation within the healthcare system would mean shifting the payment models to other systems, with new payment schemes based on processes and outcomes of care for chronic diseases, or new bundle payments where the payment would cover an episode of care or care over a defined time period.^5,17,18,32,46–51^

Based on the experience, the implementation of VBHC models has succeeded in those countries working under a per-service models, which is the case of the USA,^52^ while tax-based models can be more challenging, as it has been described in the literature.^9,17^ Despite all the efforts in different geographies and healthcare systems, the literature reveals that the execution should be undertaken by small projects and escalated based on the results, requiring substantial changes in processes and operations.^9,39,40^

### 3.6. The pioneers of VBHC in Europe

The experience of three Swedish University hospitals, which have implemented a VBHC model, was recently published. It describes the implementation on a provider level and how it varied between different areas of care with a better fit in well-defined care processes, such as elective cardiovascular care, but with substantial challenges in the care of multimorbid patients with complex needs.^39^ Furthermore, organization and internal communication were identified as important factors for the success or failure of the implementation process. In the case of the Karolinska University Hospital, a process largely led by external consultants and introducing VBHC as a completely new model rather than a gradual development of previous process-oriented work failed to gain widespread acceptance among clinicians. When administrative burden and costs, in fact, turned out to increase with no obvious improvement of quality, further implementation was halted, and the organization model was revised by embracing a less radical and theory-based approach to value-based care. By restoring the environment of traditional medical specialties without reversing patient-centred multidisciplinary collaborations, enhancing initiatives for improvements driven by employees ‘on the floor’, and a data-driven follow-up of outcomes and costs, long-term beneficial effects on the hospital´s overall performance were achieved.^39,53^

### 3.7. Challenges to adopt VBHC models in cardiology

**Figure 5:**
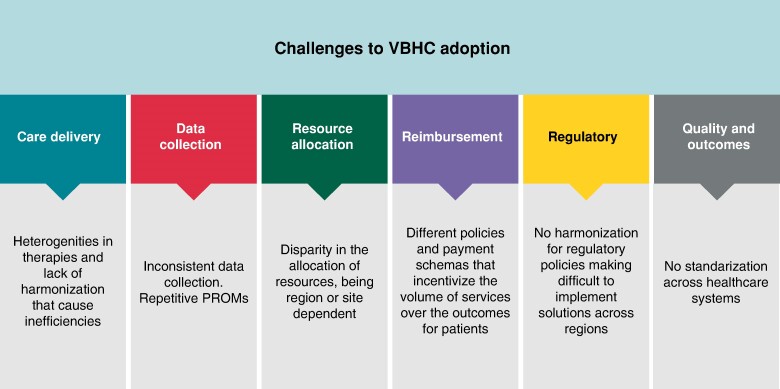
Challenges to adopt value-based healthcare (VBHC) models.

Although there are numerous benefits of the implementation of VBHC models and there is a global desire to improve societal wellbeing through these new healthcare models, in the present landscape, there are some challenges that slow down the deployment of VBHC. These could be summarized in six major points (*Figure 5*).^18^ Inefficiencies in the healthcare delivery, which according to the literature, lacks standardization and presents a lot of *heterogeneity in cardiac care and arrhythmia* therapies.^18,46,48^ The *inconsistency in the data collection* and reporting, with an absence of standards that complicates the comparison of data across healthcare systems and interoperability of tools.^54^ The *disparities across resource allocation* where some regions might overinvest in certain areas while underinvest in others. Some noticeable examples of these disparities are the differences between the resource allocation in rural and city areas. Or the differences between mature economies and developing countries, where mature economies have payment systems tied into value, while the second ones are struggling with basic needs such as coverage and access to healthcare.^6,17,39^  *Complexity in the reimbursement and payment models*, with variability in the financial structures across the healthcare systems^19,45,46,55^ and incentive programs not aligned with the goals of VBHC, especially in geographies that have not undergone a complete transition into the VBHC model.^10,52^ The great complexity in *regulatory and policy*, where different healthcare policies across regions or countries pose a challenge to comply with multiple sets of rules.^18^ And the *variability in quality and outcomes*, where the noticeable lack of harmonization and standardization across different healthcare systems creates disparities at the time of measuring the quality outcomes in the cardiovascular environment.^56^

## 4. Discussion

This review examines the concept of VBHC and its implementation in cardiology, with a focus on arrhythmia therapy. While VBHC offers a promising approach to improve patient outcomes while reducing healthcare costs, our findings reveal several challenges acting as an impediment to the standardization of VBHC models in the healthcare system and cardiology in particular. These challenges have significant implications for the successful adoption of VBHC practices.

One major challenge identified in this review is the lack of harmonization and consensus on the quality metrics and clinical outcomes. The entire VBHC model relies on the ability to measure and report specific patient outcomes. Nevertheless, our review of the literature underscores the existing variations in metrics and the absence of standardized reporting systems. This is a major impediment to making direct comparisons across the different arrhythmia units and therefore assessing the true value delivered.^16^

Furthermore, the use of health information technology presents another challenge. While electronic health records (EHR) and telehealth solutions hold great promise for improving care coordination, data accessibility, and data analytics, the lack of interoperability across different information technology systems remains a significant barrier. The incorporation of new technologies, such as mobile apps or mobile health (mHealth) solutions, represents the practice of medicine and public health with the support of mobile devices. This technology adoption into clinical workflows in the last few years necessitates substantial reform of the existing healthcare systems. This practice conversion is a complex and slow process.^16^

The documents supporting the review have highlighted the disparities in resource allocation across the healthcare system. These discrepancies can lead to variations in the availability and accessibility of advanced healthcare technologies and interventions, affecting the quality and cost-effectiveness of care. Achieving standardization in this environment is inherently challenging, as the standardization would presuppose equal access to care and resource allocation and the traditional healthcare systems present inherent limitations in their infrastructure and operations.^17^

While VBHC models are centred around the capacity to evaluate outcomes, it is crucial to create the necessary instruments and tools that enable promotion, monitoring, and rectification of any deficiencies, so the delivery of value in the context of cardiology is warranted.

### 4.1. Limitations

In our investigation into the implementation of VBHC practices within the cardiology department, it is crucial to note that the available literature and data resources presented very limited information on this subject. Despite our comprehensive search across various databases and consultation of relevant publications, it became apparent that experiences and outcomes related to VBHC in the context of cardiology are not extensively documented. Furthermore, the scarcity of published results raises the possibility that significant aspects of VBHC implementation, local challenges, and specific outcomes may not have been widely reported or are yet to be explored. This paucity of information underscores the need for more research and publication in the field of cardiology, and in the context of electrophysiology, with the objective to contribute valuable insights to the broader discourse on VBHC.

## 5. Conclusion

Despite the benefits for the population and the progress so far, no European healthcare system has been able to establish a VBHC at a national level, although countries such as Sweden have already started with the necessary changes for the implementation of this model.^39^

The deployment of VBHC models poses many challenges, mainly related to the existing infrastructure, policies, reimbursement schemas, and operation models in the healthcare system and, by extension, in the context of the cardiology department.^18,39^

The implementation of VBHC requires a call to action to all the stakeholders. It should be a priority to create patient-centred frameworks, focused on evidence-based approaches in accordance with the guidelines, and cost-effective and high-quality treatments adding value to each patient. This implementation should be supported by data collection, analysis, and the creation of new roles at the cardiology unit level. This shift would ensure the care pathway is well-defined for each patient and would change the existing environment towards the creation of sustainable models with better resource allocation and improved outcomes.

## Data Availability

Data is available upon request.
